# Aetiology of biliary atresia: what is actually known?

**DOI:** 10.1186/1750-1172-8-128

**Published:** 2013-08-29

**Authors:** Claus Petersen, Mark Davenport

**Affiliations:** 1 Department of Pediatric Surgery, Hannover Medical School, Carl-Neuberg-Str. 1, 30625 Hannover, Germany; 2 Paediatric Liver Centre, King’s College Hospital, London, United Kingdom

**Keywords:** Biliary atresia, Neonatal cholestasis, Animal models, Etiology, Neonatal immunology

## Abstract

Biliary atresia (BA) is a rare disease of unknown etiology and unpredictable outcome, even when there has been timely diagnosis and exemplary surgery. It has been the commonest indication for liver transplantation during childhood for the past 20 years. Hence much clinical and basic research has been directed at elucidating the origin and pathology of BA. This review summarizes the current clinical variations of BA in humans, its occasional appearance in animals and its various manifestations in the laboratory as an experimental model.

## Background

There are few diseases where so much is known yet so little understood than the condition of biliary atresia (BA). Nonetheless, as our current strategy (Kasai portoenterostomy (KPE) and liver transplantation where necessary) for dealing with BA is relatively successful and about 80-90% of currently affected infants will survive to adolescence and adulthood-does it matter that the cause in most cases is still obscure? In most probably not, although there is a still considerable risk of significant morbidity related to cirrhosis in those who have had Kasai portoenterostomy alone. About half of even biochemically normal adolescent survivors will have histological cirrhosis [1] and have the potential for decompensation, portal hypertension and even malignancy [2]. Furthermore, survivors with successfully transplanted organs still need to remain pharmacologically immunosuppressed and therefore have an increased risk of infection and again malignancy in the form of post-transplant lymphoproliferative disorders.

The key question that this review aims to address is what is biliary atresia and what may cause this rare disease? Increasingly it is evident that BA is not a single entity with a single aetiology but rather appears to be a phenotype characterized by an obliterated (or absent) biliary tree presenting in the first few weeks after birth [3].

The scope and limits of review are to collate observable evidence both from the human condition and the animal laboratory and try to stitch these two disparate views into one distinct picture.

### Biliary atresia in the clinic

**Figure 1:**
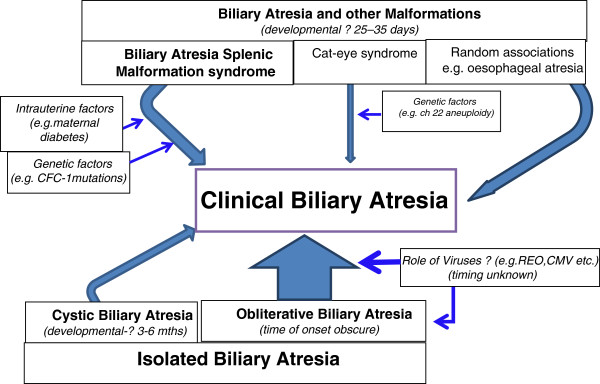
Patterns of Clinical Biliary Atresia.

Although most older textbooks usually talk in terms of only two variants, embryonic and perinatal, this is far too simplistic and it also assumes that we know a lot more about when the disease starts than we actually do [4]. We prefer to use more verifiably descriptive names with less assumption on cause. Thus, there are probably at least four clinical variants which can be defined (Figure 1).

### Biliary atresia and other congenital malformations

Firstly there are those infants with BA who have other congenital anomalies. These can be further sub-divided into three groups. Firstly there are those who have elements of the Biliary Atresia Splenic Malformation (BASM) syndrome [5,6]. These might include splenic malformation (usually polysplenia, but also asplenia and double spleen), disorders of visceral symmetry (e.g. situs inversus and malrotation), malformations of the intra-abdominal veins (e.g. absent inferior vena cava, preduodenal portal vein) and cardiac anomalies. These infants are usually girls, and some seem to come from an abnormal intrauterine environment (e.g. maternal diabetes, maternal thyrotoxicosis). It is almost certain that their bile duct pathology occurs at the same time as their other developmental anomalies (i.e. during the embryonic phase of organ development, perhaps at 5-6 weeks of gestation) and therefore at the time that the hepatic diverticulum is pushing into the mesenchyme of the prehepatic septum transversum. This is well before any intrahepatic duct system has formed (7-10 weeks) and at operation concurs with the observation that the extrahepatic biliary tree is often atrophic with little inflammation and there is usually absence of the common bile duct. Surprisingly, given this timeline the liver parenchyma at the time of birth is actually normal [7] and there is no difference in their birth weight or gestational age compared to those with isolated BA [8]. There are also clear racial differences in that this type is infrequent in the high incidence areas of the world for BA (e.g. China, Japan, Korea). Variation in BASM incidence can also be shown in multiethnic populations such as that of England and Wales [8]. It is this group which seems likely to have a clear genetic basis for their spectrum of anomalies (*pace* maternal diabetes). So of the range of possible bile duct development genes (see later); CFC-1 mutations have been have identified in 50% of a French series of infants with BASM [9]. This gene, present at Ch 2q 21.1, encodes for CRYPTIC protein and mutations have been related to heterotaxy syndromes (i.e. randomised situs) and cardiac anomalies such as transposition of the great vessels/ double outlet right ventricle [10].

There is a second group of infants, also with BA, who have other features of other distinct syndromes. The example for this is the so-called cat-eye syndrome (coloboma, ano-rectal atresia etc.) and in these chromosomal aneuploidy (Ch 22) has been shown [11].

Finally, some infants with BA have non-syndromic congenital anomalies such as oesophageal atresia, jejunal atresia, ano-rectal atresia etc. but none of the peculiar anomalies listed above and for which we have no convincing genetic explanation.

### Cystic biliary atresia

The extrahepatic component of BA is usually characterised by atrophy or absence (particularly in BASM) or by inflammatory obliteration of an intact tree. However, in about 10% of cases, cyst formation (bile or mucus) can occur, and may lead to diagnostic confusion with early obstructed cystic choledochal malformation.

Cholangiography at surgery invariably shows that the intrahepatic ducts are grossly abnormal with irregularity and pruning if there is preservation of a tree-like pattern or a cloud-like appearance caused by multiple interconnections of filamentous intrahepatic biliary ductules. Such infants belong within the BA spectrum family and should be termed cystic biliary atresia (CBA) [12]. These don’t seem to have any racial, genetic or epidemiological peculiarities but what is clear is that they are observable on antenatal ultrasound scanning–if looked for. In our experience about half are detectable–usually the large ones–and from at least 18-20 weeks gestation [13]. They also have a better outcome following surgery, probably because of better quality of luminal continuity with those intrahepatic ducts.

### Isolated biliary atresia

This is the largest clinical grouping and epidemiologically there is wide geographical (and presumed racial) variation across the world from about 1 in 5000 births in Taiwan to about 1 in 18,000 in Northern Europe with no overt seasonal variation and an equal gender split [8,14-16].

So what do we know of their aetiology? The initial hypothesis was that these infants also had a developmental problem of their bile ducts. The timing of onset must be later than the syndromic groups as no other system appeared abnormal and perhaps at one of the key stages in biliary development–the juxtaposition of the intrahepatic and extrahepatic bile ducts. Both elements have different origins, develop along separate lines from different structures and biliary continuity is only established by 10-12 weeks gestation. Tan et al. compared the appearance of serial sections at the level of the porta hepatis in normal fetuses at this gestation with the histological appearance of the porta of infants with established isolated BA [17]. They were remarkably similar showing multiple biliary ductules around branches of the portal vein undergoing selection, deletion and then morphing into the larger fully-formed ducts by 13-14 weeks. So, the hypothesis suggests that even isolated BA might be a form of 1st trimester arrested development–for whatever reason.

The alternate hypothesis is that such infants once had a completely formed intact biliary tree but that obliteration occurs as a secondary, indeed perinatal, phenomenon [18,19]. The clinical evidence supporting a patent bile duct system and then obliteration is difficult to obtain. Anecdotal reports of an initial “neonatal hepatitis” picture on investigation but evolving into biliary atresia later have been suggested. Postnatal biliary obliteration can certainly happen and we previously described 3 infants who became jaundiced secondary to perinatal events (proximity surgery and probable healed bile duct perforations). Their extrahepatic bile ducts were completely obliterated although they retained a normal intrahepatic biliary tree which had dilated by the time of definitive surgery [20].

Amniotic fluid has been assayed for the hepatic-exclusive enzyme γ-glutamyl transpeptidase in one large screening study. The lowest values were found in 3 infants who ultimately proved to have BA which clearly implied a prenatal onset and absence of bile from the fetal gastrointestinal tract [21]. The level of bile acids in the Guthrie blood spots (taken in first few days of life) has also been measured [22] and showed that 47 (77%) of 61 infants who later proved to have BA had elevated total bile acids (>97th percentile, 33 μmol/L) with no difference if the blood had been taken at <7 days or >10 days. This suggests that cholestasis is already obvious (if looked for) during the first post-natal week in at least three-quarters of those who prove to have isolated BA. A similar North American study of split bilirubin levels in 31 infants obtained when they were < 48 hours old and who later turned out to have BA also showed levels distinctly higher than controls [23], again implying established biliary pathology at the time of birth.

Our last, and perhaps most controversial clinical variant has been that of viral-associated BA–which some might say is simply the latter group plus a culprit caught in the act. The role of viruses in the animal model (whether as instigators or genuine pathogens) will be outlined in detail later but clinically this entity has been the subject of much debate since the 1980s and 90s. Initially, work focused on serology of REOvirus type 3 [24], but a more sophisticated search evolved for actual viral footprints within the liver and specifically for group C rotavirus (positive in 50% of BA infants) [25] and cytomegalovirus [26,27]. Most recently our Hannover group [28] examined liver biopsies in 74 infants with BA against a panel of possible hepatotropic viruses and showed that viral RNA/DNA could be found in just less than half (REOvirus 33%; cytomegalovirus 11%; adenovirus 1% and enterovirus 1.5%). The debate revolves around whether these are simply (not so) innocent bystanders or integral to the cholangiodestructive mechanism. The faecal flora of infants with BA is different [29] and enteric absence of bile salts may allow selection of such enteroviruses. Does it make a difference? In the follow-up to the German study there was no difference in outcome between virus + ve and virus–ve infants[30]. However, in a separate study using only infants who tested IgM + ve for CMV (n = 20) from our London group compared to 111 infants with negative IgM serology we have shown reduced clearance of jaundice and a significantly increased mortality in the CMV IgM + ve group [31].

### Cellular kinetics, inflammation and clinical biliary atresia

There is a potent inflammatory reaction in the livers of infants with isolated BA, focused on the portal tracts [32-34]. There is abnormal expression of MHC Class II antigens and the adhesion molecules ICAM and VCAM on both vascular and biliary endothelium, together with a sometimes dense population of activated and proliferating mononuclear cells [35], [36]. Among these are mast cells [37] and Kupffer cells [38] but they seem to be predominantly CD4^+^ T cells and NK cells [39,40] orchestrating a Th1 cytokine response [41,42] with increased expression of IL-2, IFNγ, TNFα and IL-12 and other proinflammatory factors, e.g. iNOS and NO [43,44]. The cell lines appear to be oligoclonal at least in the CD4^+^ and CD8^+^ subsets identified by Mack et al. [45]. Alternatively, Shinkai et al. observed a predominantly CD8^+^ infiltrate in their infants with BA [46,47] though their study group was much smaller; and previous work has suggested poor CD8^+^ cytotoxic function [48]. Muraji et al. has also described significantly more CD8+ T cells in the livers of BA infants, and interestingly, these appear to be of maternal origin [49]. This is the interesting concept of *maternal microchimerism*, whereby transplacental passage of immune active cells (seemingly CD8^+^ CD45^+^) occurs, localizing within the liver and initiating a form of graft-versus-host reaction. Such cells may also presumably provide an alternative target for an autoimmune response. Finally, a third subset of helper T cells, Th17^+^ cells also seem to play a significant role in mucosal host defence and have been implicated in some autoimmune cholangiopathies such as primary biliary cirrhosis in adults. Such Th17^+^ cells, can be shown to accumulate at the bile duct epithelial interface and infiltration of this subset in portal tracts and also appears to be a characteristic of BA [50,51].

There is also a pronounced soluble inflammatory component observable at the time of presentation. Initially this with increased levels of sICAM and sVCAM [52,53] and the cytokines IL-2 and Il-10 compared to controls which then actually increases following the KPE. In the case of IL2, IL-18 and TNFα this increase is very marked to about 9-12 months. If there has then been resolution of jaundice then it tends to abate but only sICAM appears to be closely correlated with plasma bilirubin levels [54].

Early-onset of liver fibrosis appears to be a defining characteristic of BA, perhaps not seen in other cholestatic conditions of infancy such as Alagille’s syndrome or α-1-antitrypsin deficiency. Harada et al. cultured human intrahepatic biliary epithelial cells (BECs) and showed that exposure to a viral RNA analogue triggered an immune reaction which persisted long after removal of the virus [55]. Excessive activation of Hedgehog (Hh) signaling pathways and increased expression of matrix metalloproteinases (MMP) [56] can be shown in BA infants promoting epithelial–to–mesenchymal transition of BECs and leading to enhanced hepatic fibrogenesis. Resident (Kupffer cells) or systemic / recruited macrophages /monocytes seem to play a dual role in this process. Initially they may act as antigen presenting cells (APCs) early on in the process but they also appear to be the key factor in this development of fibrosis. Tracy et al. in 1996 first highlighted the increase in the resident macrophage population in BA and increased levels of both CD68^+^ cells and its circulating markers (TNFα and IL-18) have been shown [57,58].

### Biliary atresia in the animal world

The lamprey is an eel like creature which parasitizes and feeds on the blood of fish and is unique amongst vertebrates in that its liver does not have a gallbladder, biliary tree or even canaliculi. It does have them in the larval stage but then they disappear on metamorphosis to the adult stage. The only real effect on their hepatocytes appears to be increased numbers of cytoplasmic vacuoles presumably where some form of bile degradation occurs [59,60]. However, despite a series of papers, which looked at the lamprey as a potential animal model for the congenital form of BA, this direction is no longer pursued.

There have been sporadic reports in the veterinary literature of biliary atresia or at least BA-like lesions in lambs, foals, dogs, calves and even a Rhesus monkey (associated with REOvirus) [61,62]. An amazing epidemic of BA in over 200 lambs and 9 calves was attributed to maternal grazing and ingestion of a toxic weed known as the red crumbweed (*Dysphania glomulifera subsp. glomulifera*) growing on the newly exposed silt foreshores of Burrinjuck Dam, New South Wales, Australia [63].

### Biliary atresia in the laboratory

#### Bile duct development

There is much ignorance about the steps of bile duct development during the embryonic period, and what is known has been largely derived from work in mice and zebrafish [64] and is somewhat outside the remit of this review. The “ductal plate” refers to differentiation of hepatoblasts into BECs and then condensation around in-growing portal venules starting at the porta hepatis and occurs at around 8-10 weeks with later remodeling and definitive tubularisation via selection and deletion. This appears to be induced in the fetal liver by a periportal gradient of activin/transforming growth factor-beta (TGFβ) signaling. The current consensus is that the entire intrahepatic system is derived from this mechanism as suggested by Desmet [65]. The establishment of biliary continuity with the lumen of an already established foregut diverticulular structure occurs although when and how is indistinct. The hepatocytes start to secrete bile from about the 12th week.

Cholangiocyte specification is promoted by the Wnt/β-catenin signaling pathway and initiation and overall control appears to be a function of the Notch signaling pathway, Notch 2 rather more so than Notch 1 [66,67]. Certainly, mutations in the latter’s ligand (Jagged1) appear responsible for bile duct hypoplasia of Alagille’s syndrome (and murine equivalents). Various transcription factors (e.g. Hes-1, Hnf-6, Foxa1/2, TGF-β) are also involved in remodeling of the ductal plate and prevention of excess cholangiocyte proliferation.

Inversin (Inv) is a gene localised to Ch 4 in humans. The inv mutant mouse, created by insertional mutagenesis, was first reported in 1993 by Yokoyama et al. [68], who described situs inversus and severe jaundice in homozygous mutants. The actual biliary pathology has been disputed however, with Mazziotti et al. [69] suggesting extrahepatic discontinuity but more recently Shimadera et al. [70] showing periportal ductular proliferation (perhaps akin to ductal plate malformation) and anomalous development of the intrahepatic bile ducts but a normal extrahepatic system. Neither group demonstrated inflammatory, ischemic or cystic features.

#### Inducing cholestasis and atresia in prenatal experiments

Ligation of the common bile duct in fetal lambs at about 80 days of gestation produces cholestasis and cystic change in the bile duct evident at birth and, perhaps, resembling those infants with CBA [71,72]. Similar findings can be shown in fetal rabbits when the hepatic artery has been ligated and in one study absence or hypoplasia of the intrahepatic bile ducts was also seen in 5 out of 14 five-week old pups [73].

The effect of drugs administered during pregnancy has also been studied. Hosada et al. administered intraperitoneal phalloidin, an actin-binding toxin derived from the Death Cap mushroom (*Amanita phalloides*) to Wistar rats on gestational day 15. After birth, the pups presented with cholestasis and an increased volume of actin filaments around bile ducts, but without typical BA features [74]. In another study, the anti-helminthic drug, 1,4-phenylenediisothiocyanate, was given orally to pregnant, newborn and adult Wistar rats. Peribiliary inflammation and dilatation was found in the post-natal drug groups but the most convincing BA-like findings with almost complete obliteration but no dilatation was found in those exposed to the drug during the fetal period and at one month after birth [75].

#### Inducing cholestasis and atresia in postnatal experiments

A number of manoeuvres, e.g. ligation of common bile duct, intrabiliary injection of sclerosants and superglue, in a variety of animal models, e.g. mice [76], rat [77], and minipigs [78], have been reported with the intention of reproducing early neonatal cholestasis. These studies seem to have more value in replicating the metabolic consequences of cholestasis in this age-group rather than providing a real insight into aetiology. One exception may be that of Schmeling et al. [79], who infused phorbol myristate acetate (PMA) into the gallbladders of golden hamsters. PMA is not in itself toxic but it does activate peribiliary neutrophils which release potent free-radical oxygen species thereby setting up a marked peribiliary inflammatory process and ultimately fibrosis. Actual obliteration and the effects on the extrahepatic biliary tree were not commented upon.

A possible immune-mediated mechanism was first investigated by Schreiber et al. in 1992 [80]. They transplanted fetal, neonatal and adult bile ducts taken from C57BL/6 into the adult B10 mice and observed a dynamic pattern of rejection and fibrosis. They found that prenatally harvested bile ducts were relatively “immunoprivileged” because of diminished expression of HLA class I and II antigens and that in adult mice, graft rejection could be stopped by cyclosporin A.

#### The viral model of biliary atresia

Initial mice models used intraperitonal inoculation of REOviruses with varying serotypes, e.g. type 3 (Abney) [81,82]. All seemed to induce liver and biliary inflammation with jaundice as a consequence, but without real features of atresia.

Oral administration of REOvirus to newborn ND4 Swiss Webster mice showed that hepatotropism was higher in those strains which have the capacity to bind sialic acid as a co-receptor. These mice presented with oily fur syndrome, portal lymphoid infiltrates in the liver and sporadic bile duct necrosis. However, mortality appeared to be due to encephalitic disease, and surviving pups recovered completely from their hepatobiliary pathology [83]. Similar observations were made when other hepatotropic Rotavirus and REOvirus strains were used [84,85].

**Figure 2:**
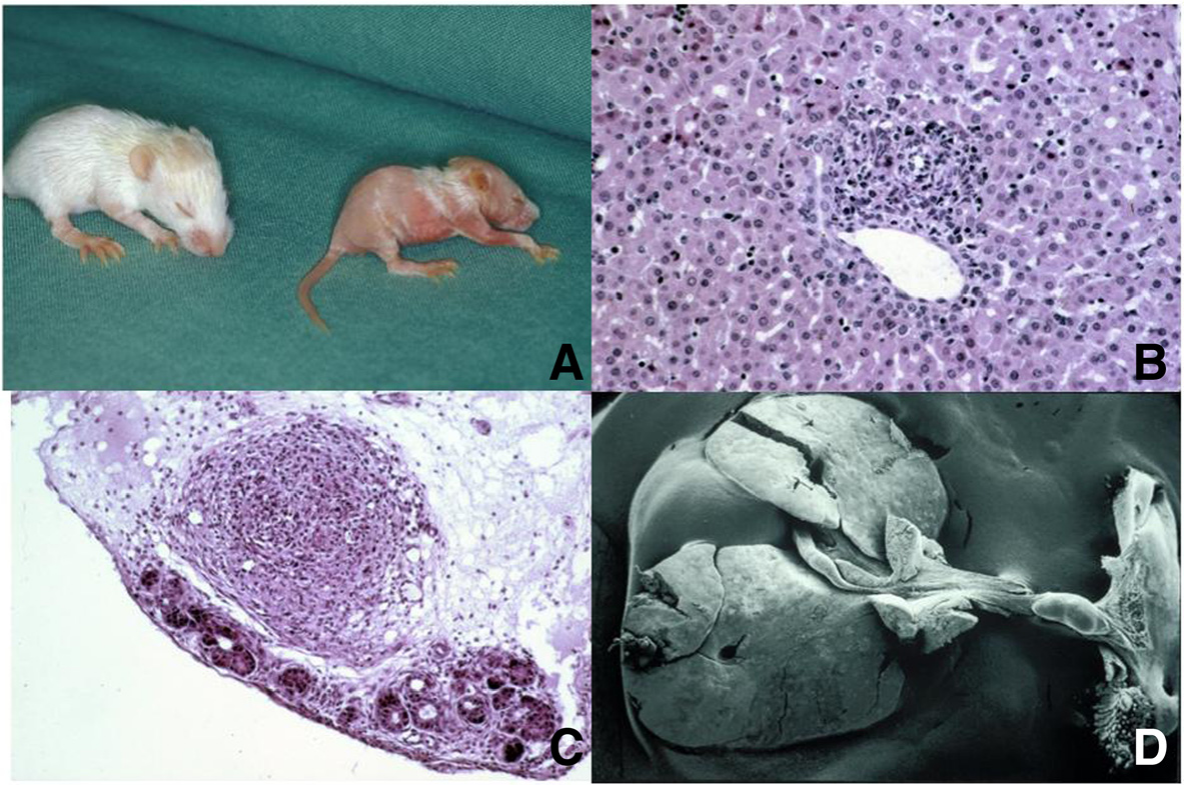
**The Rhesus Rotavirus induced biliary atresia model in newborn Balb/c-mice. (A)** Two 14 day old Balb/c mice, left, healthy control, right, jauniced with oily fur syndrome. **(B)** inflammatory infiltration in a portal field (9 day old, H x E 400×). **(C)** Section of the hepatoduodenal ligament at the level of the common bile duct atresia (16 day old, H x E 250×) (59). **(D)** Scanning electron microscopy of a whole specimen, showing a small gallbladder, a vanishing common bile duct along with the hepatoduodenal ligament a prestenotic cystic dilatation near to the duodenum (15 day old) (56).

A better model with peribiliary inflammation leading to features of actual irreversible extrahepatic atresia was developed by infecting newborn Balb/c-mice with Rhesus Rotavirus (RRV) [86]. In the initial study [87] about half of the orally RRV infected pups developed cholestasis, and most died within 3 weeks. Dissection showed obstruction of the common bile duct and BA-like changes in the liver, e.g. inflammatory infiltrates and bile duct proliferation. Our Hannover group modified this model using intraperitoneal RRV given at 24-48 hours with an increase in the proportion becoming cholestatic and the development of atresia-like biliary segments (Figure 2) [88-90]. Still, older 3 week old pups failed to show much in the way of liver fibrosis, and using picosirius red staining collagen deposition around portal triads was much less obvious than in the human condition [91,92].

To work, however, three preconditions must be fulfilled; but it offers a way of understanding a mechanism for the observable pathology. The preconditions are such that the strain of mouse, the timing and the dose of virus inoculation must be perfect to achieve BA-like pathology [89,93,94]. Maternal infection during late pregnancy does not induce jaundice, but does prevent the offspring from developing cholestasis following postnatal RRV application [95,96]. Post-natal inoculation at 12-24 hours after birth produces BA-like lesions in over 90% of animals compared to less than 20% if the inoculation is given at 48-72 hours. Table 1 illustrates this effect of mouse strain time of virus administration and viral load on outcome, and shows that too high a dose is lethal before the onset of biliary pathology. On the other hand, changing of the RRV-dosage or the mouse strains, respectively, does not necessarily prevent infected pups from cholestasis, but from developing destruction and atresia of extrahepatic bile ducts. Those mice mostly catch up weight and recover completely. The versatility of the model is also limited as sequential investigations cannot be performed in the same animal. Diseased pups are extremely unstable and too small for repetitive biopsies or blood sampling. However various stages of the assumed immune cascade can be studied by extraction of cell lines, e.g. cholangiocytes taken from RRV infected pups can be isolated [97-99]. One advantage of this model is that modification of each of the preconditions allows investigation of subsequent immunological or cellular changes.

**Table 1:** The incidence of virally induced cholestasis and biliary atresia depends on three variable parameters, mouse strain, time (hours post partum) and dosage of Rhesus rotavirus application

**Variable parameter**	**Cholestasis**	**Biliary atresia**
**Mouse strain**		
Balb/c ^55, 71^	67%-85%	67%-91%
CD ^59^	33%	46%
NMRI ^59^	19%	50%
C57Bl6 ^67^	13%	100%
Balb/c-IFN-γ–/–^65^	90%-100%	20%
Balb/c-TNF-α–/–^68^	No data	86%
Balb/c-Mx + −A2G ^71^	65%	65%
WT 129 ^70^	30%	50%
A 129 IFN-α/β receptor–/–^70^	79%	96%
G 129 IFN-γ receptor–/–^70^	39%	86%
AG 129 IFN-α/β/ γ receptor–/–^70^	70%	96%
**Age at infection**		
12-24 hours ^65, 83^	80%-86%	90%-100%
24-48 hours ^55^	61%-85%	69%-91%
48-72 hours ^52, 53^	13%-42%	0-17%
**Infective dose**		
10^7*^ pfu ^62^	100%	100%
10^6^ pfu ^62^	86%	100%
10^5^ pfu ^62^	38%	100%
10^4^ pfu ^62^	0	0

pfu–plaque forming unit.

* >10^6^ pfu is not recommended, because the early lethality is inacceptably high.

The dynamic pattern of RRV infection can be monitored on the basis of the expression of mRNA encoding RRV non-structural and structural proteins, e. g. VP6, VP4, and viral plaque assays. So, the peak of viral load is seen at day 7 after inoculation, and is mostly cleared from the liver by day 14 [97-99]. However, the cellular and humoral immune response persists even after complete viral clearance.

**Figure 3:**
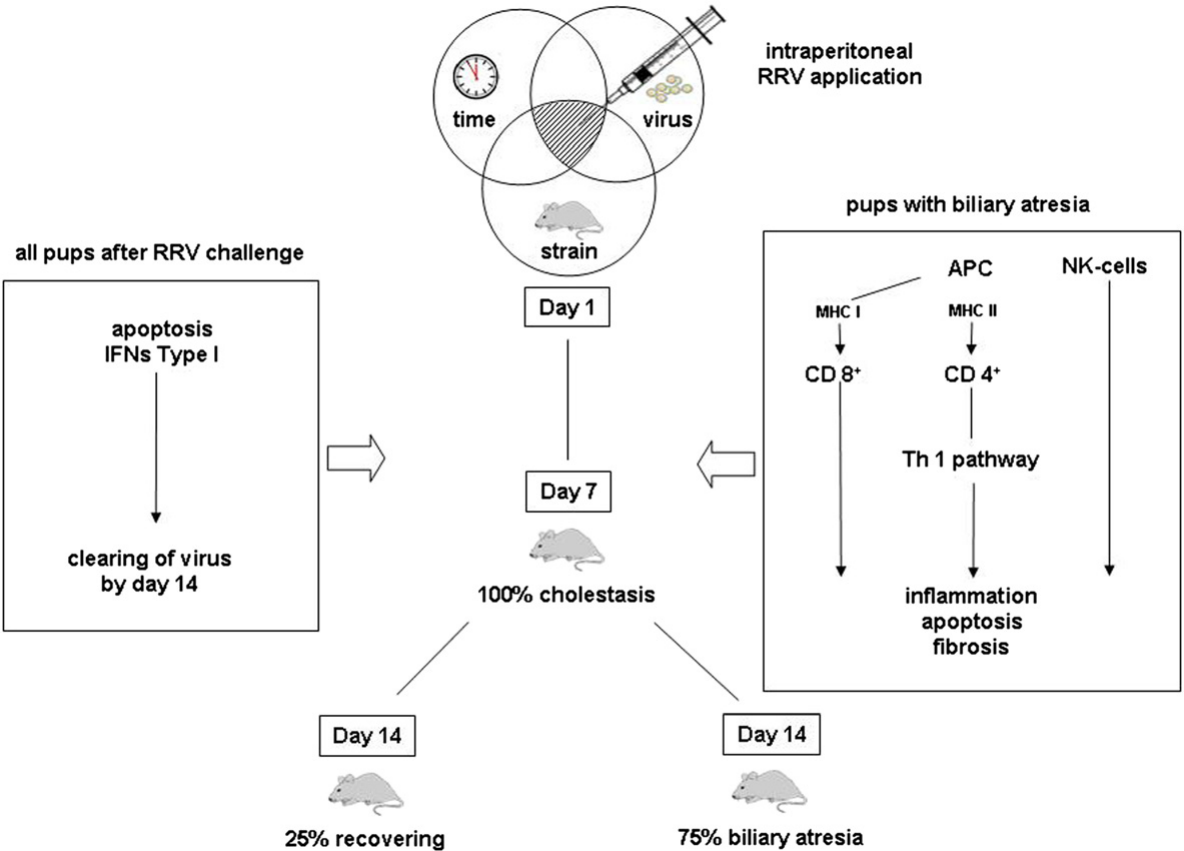
**Schematic network of cellular dynamics and immunological cascade in mouse model of biliary atresia.** For more details see Table 2.

The steps of the immune reaction to RRV inoculation appear to be as follows (Figure 3). The viruses target cholangiocytes and macrophages [100], while the initial response depends upon innate NK cells and APCs inducing a T-cell mediated immune response. In this murine model, cholangiocytes and dendritic cells [86], appear to act as the APCs via expression of both MHC class I antigens [97,98,101] (to cytotoxic CD8^+^ cells) and MHC class II antigens (to CD4^+^ T-helper cells). Activated CD4^+^ cells, driven by IL-12, produce a bewildering range of cytokines, such as IL-2, IFN-γ, IFN-α, IFN-β, CXCL 9, CXCL 10, CCL2, CCL5, Stat 1, Granzyme A and B, Osteopontin, etc. [102,103]. Lack of IL-12 does not seem to abolish this particular response, as Th1 dependent cytokines, such as IFN-γ and TNF-α, are not reduced in IL-12 deficient mice, suggesting the presence of alternative pathways [103-105]. Recently, a Th2 response has been shown to induce epithelial injury and inflammatory obstruction of extrahepatic bile ducts in the same mouse model [106]. Cholestasis becomes evident one week after infection and the infiltration of CD4^+^ and CD8^+^ cells increased three-fold with a peak on day 14.

Interferons seem to play a crucial role in this model, as Mx protein (an IFN type I specific indicator) persists in hepatocytes, bile ducts and intrahepatic endothelial cells of cholestatic mice beyond the second week after RRV infection [107,108]. In other studies, for instance with IFN-receptor knock-out mice, it can be shown that IFN type II attenuates the tropism of lymphocytes to bile ducts and is somehow imperative to the development of BA [99,109].

Depletion of CD8^+^ cells, as well as loss of IFN-γ, reduces the incidence of experimental BA. IFN-type II regulated chemokines such as Mig, IP-10 and I-Tac can be shown to peak at day 7 only in BA-developing Balb/c but not in IFN-γ–/–mice [110]. Thus in these knock-out mice, early lobular and portal inflammation with periductal infiltration by neutrophils is still seen at day 3 and is followed by bile duct proliferation. But in contrast, they did not go on to develop bile duct obstruction or atresia and indeed recovered in most cases. Administration of recombinant IFN-γ restores this detrimental consequence. We hypothesis that both IFN-γ and CD8–T cells constitute two complementary mechanisms in which IFN-γ and other Th-1 dependent cytokines serves as a molecular signal that promotes the infiltration of injured bile ducts by inflammatory cells, while CD8- T cells, together with NK-cells, trigger the transformation of an ongoing inflammatory process into fibrosis and occlusion of the bile ducts [111,112].

The role of CD19^+^ B-cells and activation of Th2 effector cells remains unclear and the expression of Th2 related cytokines such as IL-4 and IL-5 has not been uniform [99,113].

Regulatory T-cells (T-reg) seem to be involved in this auto-immune process. In mice, T-regs are absent in the newborn and become activated during the first week of life. Initially substitution of T-regs in the mouse model was supposed to reduce the incidence of BA and shift the focus onto NK-cells [114,115]. Adoptive transfer of T-cells from mice with RRV-induced BA to SCID recipients results in periductular inflammation, and a bile duct epithelia specific T-cell mediated autoimmunity [116]. The hypothesis that humoral autoimmunity plays some role in the pathology of BA [117] has been endorsed by detection of anti-α-enolase autoantibodies in both experimental and human BA and found in patients with typical autoimmune liver disease, such as autoimmune hepatitis and primary sclerosing cholangitis.

Apoptosis of the biliary epithelium (as shown by TUNEL assay and expression of caspase 1 and 4) and activation of the complement cascade appears to peak at day 7 and can be induced by proinflammatory cytokines such as TNF-α and IFN-γ [109]. Additionally, NF-κB, which is a first responder to harmful cellular stimuli as a rapid-acting primary transcription factor inducing apoptosis, shows an increasing level from day 5 with a peak at day 15 [84,118]. It still remains unclear if apoptosis is part of the clearing mechanism after viral infection or a hyper-responsiveness consistent with immature immunity [119].

There have also been attempts to modify pathology using this model. For instance, repeated administration of IFN-α at onset of jaundice, has been shown to ameliorate the development of BA-like lesions and increase survival. It remains unclear if this boosts viral clearance or modulates the immune response [120,121]. Immunisation of dams with RRV before mating and during pregnancy protects their offspring from cholestasis and development of BA [95,96]. Dams, which were preconceptionally and orally vaccinated with commercially available rotavirus vaccines, did not prevent their RRV infected newborns from becoming jaundiced, but did prevent the development of BA [122]. While these preliminary results are far from clinical application in humans, the potential for at least some form of prophylaxis has been shown.

Table 2 illustrates a chronological overview of the key immunological observations mentioned in the text in both human and experimental BA to date.

**Table 2:** All papers, which have so far been published concerning BA and immunology in both, human and experimental BA

**Effector**	**Subset/Cytokine**	**Human (Reference)**	**Murine (Reference)**	**Key Message**
T-regs		[123]		Circulating and local T-regs in lymph nodes of the porta hepatis are reduced in CMV positive patients with BA, compared to age matched controls with other cholestatic liver diseases
CD4 + −T-Cells	Osteopontin	[43]	[124]	Osteopontin mRNA expression was shown in liver biopsies of BA patients and also in experimental BA
Cholangiocytes	TLRs	[125]		TLRs 2 and 8 mRNA expression was higher in early states of BA-patients and significantly elevated to age matched controls
CD4 + −T-Cells	Th_2_/IL-13	[106]	[106]	STAT1–/–mice infected with RRV exhibit a Th_2_ dominated inflammation, evidence can be found in humans, too, also mediating experimental biliary atresia if not accompanied by IL-13 blockade
CD4 + −T-Cells		[41]		DNA-Hypomethylation throughout the genome of CD4 + −T-Cells is negatively correlated with IFN-ɣ mRNA-levels compared to healthy controls
Dendritic Cells		[86]	[86]	RRV-primed DC, also present in human livers, boost lymphocyte expansion and mediate epithelial injury whereas depletion of DC or blocking IL-15 reduced NK-Cell activation
Macrophages	Mip2/Cxcl2		[100]	RRV-infected Macrophages influence neutrophil chemotaxis
	iNOS		[44]	Liver samples show a strong correlation between NF-κB -activation and iNOS hyperexpression
T-regs			[114,126]	Activated T-regs reduce inflammatory cytokine production and suppress NK-cell activation in vitro and in vivo
Cholangiocytes			[101]	Release of CXC–and CC-Chemokines by cholangiocytes markedly increase upon onset of disease
Cholangiocytes			[97]	In the setting of RRV-infection and Th_1_ inflammation cholangiocytes produce inflammatory cytokines and chemokines but do not function as APC despite expressing all necessary surface markers
NK-Cells		[127]	[115]	NK-Cells populate the liver of mice, and humans, and their depletion or blocking Nkg2d prevents cholangiocyte lysis in vitro and in vivo
	IFN-ɣ /TNF-α		[109]	Inhibition of caspases reduces apoptosis induced by synergism of IFN-ɣ and TNF- α
CD8 + −T-Cells		[49]		Primarily CD8 + −T-Cells of the lymphocyte infiltrate suffer from maternal micochimerism
	NF-κB gene products	[55]		Intrahepatic Biliary Epithelium reacts with NF-κB activation if treated with a viral-dsRNA-analogon
	TNF-α		[103]	Although highly elevated in experimental biliary atresia blocking TNF- α exerts no effect
T-Cells		[45]		CD4+ and CD8 + −T-Cells show oligoclonal expansion of TCR Vβ with Vβ20 dominating in the latter
	NF-κB gene products	[117]		Biliary Epithelium reacts similar to a viral infection if treated with a viral-dsRNA-analogon
CD8 + −T-Cells			[110]	Depletion of RRV-primed CD8 + −T-Cells reduces disease incidence and mediate the epithelial injury
	IL-12		[105]	Loss of IL-12 in mice does not prevent experimental biliary atresia but shifts the Th_1_-phenotype of inflammation towards neutrophils
CD4 + −T-Cells	IFN-ɣ		[116]	Although not being able to elicit experimental biliary atresia in SCID mice RRV-primed CD4 + −T-Cells are the source of IFN-ɣ
	IFN-ɣ		[107]	IFN-ɣ-RII is without influence on development of experimental biliary atresia
CD8 + −T-Cells		[46]		Elevated numbers of CD8 + −T-Cells populate the portal tract in biliary atresia
T-Cells		[47]		T-Cells within the livers of diseased express more CXCR3 than controls
	IFN-ɣ		[128]	RRV-challenge of mice upregulates Interferoninducers followed by the IFN-ɣ network genes with onset of disease in the biliary transcriptome
CD4 + −T-Cells Macrophages	Th_1_ TNF-α		[113]	With RRV present one week p.i. the portal tract infiltrate is predominated by CD4 + −T-Cells producing IFN-ɣ and Macrophages producing TNF-α, whereas both effectors produce TNF-α after virus clearance
Macrophages		[129]		Increased infiltration of Macrophages in the liver after Kasai is associated with a favourable outcome
	IFN-ɣ		[99]	IFN-ɣ depletion prevents bile duct obstruction while administering IFN-ɣ to IFN-ɣ–/–induces experimental biliary atresia after RRV-injection
CD4 + −T-Cells	Th_1_	[33]		The portal tract inflammatory response is dominated by CD4 + −T-Cells with a Th1-cytokine response and an increased number of Kupffer cells
CD4 + −T-Cells		[130]		The reduced number of naïve-CD4 + −T-Cells in patients persists after transplantation
	Costimulatory factors	[35]		Costimulatory factors of APC are highy expressed on bile duct epithelium
Lymphocytes		[36]		Leukocyte infiltration of livers often do not include immunocompetent lymphocytes
	Th_1_	[43]		Within the transcriptome of pooled mRNA genes pointing towards a Th_1_-profile are often upregulated
Lymphocytes		[40]		Activated and proliferating lymphocytes populate the liver
CD8 + −T-Cells		[48]		CD8+ T-Cell infiltrate of the proliferating bile ducts after Kasai operation lack the phenotype of activated cytoxic lymphocytes
CD4 + −T-Cells		[42]		A decreased number of naïve-CD4 + −T-Cells is accompanied by reduced receptor density
Mast cells		[37]		The number of intrahepatic mast cells negatively correlates with liver function
Macrophages	IL-18	[38]		Proliferation of Kupffer-Cells is found in the liver accompanied of elevated IL-18 levels in serum
	IFN-α		[121]	IFN-α prevents experimental biliary atresia
Macrophages		[58]		Increased infiltration of Macrophages in the liver after Kasai is correlated with a bad prognosis
Macrophages		[57]		Macrophages coexpress CD68 and CD14 in biliary atresia
Lymphocytes		[34]		Lymphocyte infiltration into biliary epithelium is quite similar to GVHD
Mononuclear cells		[131]		Mononuclear infiltrate in BA is more similar to normal livers than to those suffering from chronic infection or autoimmunity
T-regs			[114,126,132]	The RRV-induced murine model of BA is associated with defects in the production and function of T-regs, probably suppressing DC-dependent activation of naive NK cells

References are listed in reverse chronological order and the key message of each paper is summed up as well as the key effector cells and/or corresponding cytokines.

The key question, of course, is does this experimental model of BA in mice really mirror the human disease and can the putative mechanism really be applied outside of the mouse [16,41]? One problem is that cholestatic pups do not survive longer than 21 days. About 80% of the RRV infected newborn mice develop jaundice by the 7th day, stop gaining weight and die within three weeks. Although lethality is 100%, the actual cause of death is still unclear, in as much as these mice no longer have the virus. It is likely that dams no longer feed their diseased pups and one task would be to improve the survival of diseased pups, perhaps by means of artificial nourishment, in order to simulate better the natural history of BA. One final fundamental dissimilarity between murine and human BA is that mice do not develop severe liver fibrosis.

Optimizing the infective mouse model is a promising outlook for understanding of the pathological mechanism in mice but it needs to be balanced by effective translational research. We first see infants with BA when a good deal of the damage has already been done and do not know whether their immune response is primary and etiological or simply a secondary phenomenon.

## Conclusion

Donald Rumsfeld served as the American secretary of state for defense from 2001-6 and highlighted rather a neat philosophical principle, although it was widely derided at the time. He said: “There are known knowns. These are things we know that we know. There are known unknowns. That is to say, there are things that we know we don’t know. But there are also unknown unknowns. There are things we don’t know we don’t know.” In terms of BA what do we know? We know it is an exceptionally rare disease and its onset is confined to the newborn period. We know that there are distinct clinical variants and therefore more than one mechanism for obliteration. Primary failure to develop luminal continuity seems obvious for “genetic” conditions such as BASM and similarly some local fetal insult or event perhaps causes CBA. What little clinical evidence there is suggests that most of the remainder are cholestatic in the first week of life. What are the known unknowns then? We struggle to relate what the best available experimental model tells us about the pathological mechanism evident in the human condition with any degree of consistency or accuracy [40,45,54]. That there is an integrated, multilayered, harmonised and structured inexorable inflammatory response to something (possibly viral) is clear, and that it occurs crucially within a very narrow window just after birth also appears obvious from the mouse model. The response is aberrant and peculiar to this period and may be auto-immune in its effect–although there is no suggestion that it continues much beyond the first few months (otherwise KPE and transplanted livers would invariably fail) or has any effect on other organs. Something makes the immature biliary tract susceptible in a way that the older child’s or adult’s liver is not. But viruses are ubiquitous, and it seems that many disparate viruses fit the offender profile laid out by the experimentalists; and if their role is crucial rather than incidental then why is this disease so rare? Improved synchronicity between those model-based workers with those able to furnish clinical material would help resolve some of these known unknowns. What then of those unknown unknowns, well we shall have to wait and see as they will surely declare themselves to a future audience.

## Abbreviations

APC: Antigen presenting cells; BA: Biliary atresia; BASM: Biliary atresia splenic malformation; CBA: Cystic biliary atresia; CCL: Chemokine ligand; CCR-5: C-C chemokine receptor type 5; CD: Dendritic cell; CMV: Cytomegalovirus; CXCL: C-X-C chemokine ligand; dsRNA: Double-stranded RNA; Foxa 1: Forkhead box protein A1; Hh: Hedgehog; GVHD: Graft-versus-host disease; Hes-1: Hairy enhancer of split 1; HNF-6: Hepatocyte nuclear factor 6; HLA: Human leukocyte antigen; IF-10: Interferon inducible protein-10; IFN: Interferon; IL: Interleukin; iNOS: Cytokine-inducible mitric oxide synthase; MHC: Major histocompatibility complex; MIP2: Macrophage inflammatory protein; MMPs: Matrix metalloproteinases; mRN: Messenger ribonucleic acid; NF-κB: Nuclear factor kappa-light-chain-enhancer of activated B cells; NK-cells: Natural killer cells; PMA: Phorbol myristate acetate; RRV: Rhesus rotavirus; SCID: Severe combined immunodeficiency; SERPINA: Serpin peptidase inhibitor; STAT: Signal transducer and activator of transcription; TCR: T-cell receptor; TGF-β: Transforming growth factor beta; TNF: Tumor necrosis factor; T-reg: Regulatory T-cells; TUNEL: TdT-mediated dUTP-biotin nick end labeling; VP6: Viral protein 6.

## Competing interests

The authors declare that they have no competing interests.

## Authors’ contributions

CP and MD designed and wrote the paper, contributing equally to this work. Both authors approved the final version of the manuscript.

## Disease name/synonyms

Biliary Atresia (BA), ORPHA30391, OMIM 210500.
